# Assessing aerobic physical efficiency through temple surface temperature measurements during light, heavy exercise, and recovery

**DOI:** 10.1038/s41598-023-43012-1

**Published:** 2023-09-23

**Authors:** Agnieszka Danuta Jastrzębska, Rafał Hebisz, Paulina Hebisz

**Affiliations:** Department of Physiology and Biochemistry, Wroclaw University of Health and Sport Sciences, 51-612 Wrocław, Poland

**Keywords:** Cardiovascular biology, Circulation

## Abstract

The study was conducted to determine thecorrelation between the selected measures of aerobic physical efficiency and changes in the temple surface temperature in response to light and heavy exercise. 25 physically active men aged 19–25 were recruited for the study. They performed a graded exercise test on a cycle ergometer to measure maximum power (Pmax) and a test verifying the value of maximum oxygen uptake (VO_2_max). Then, two 3-min submaximal efforts with constant-intensity of 2.2 W·kgLBM^−1^ and 5 W·kgLBM^−1^, respectively were performed. During the constant-intensity efforts, the temperature of the temple surface was measured. Then, the difference between the temperature of the temple measured at the end of the exercise and the temperature measured at the beginning of the exercise was calculated (ΔT1-2.2, ΔT1-5, respectively). It was shown that ΔT1-2.2 correlated statistically significantly with VO_2_max (ml·min^−1^·kg^−1^) (r = 0.49; *p* = 0.01) and Pmax (W·kg^−1^) (r = 0.41, *p* = 0.04). Moreover, ΔT1-5 correlated statistically significantly with VO_2_max (l·min^−1^) (r = − 0.41; *p* = 0.04). Changes in body surface temperature in response to light exercise positively correlate with measurements of aerobic physical efficiency, such as VO_2_max and Pmax. When the exercise intensity is high (5 W·kgLBM^−1^), the correlation between exercise body temperature changes and VO_2_max becomes negative.

## Introduction

Cardiovascular fitness assessment is used, among other things, to predict the risk of early mortality due to cardiovascular failure^1^. The size and nature of the cardiovascular response to physical effort depends on the type of effort undertaken and the conditions in which the effort is undertaken. Numerous publications concern the reactions of the circulatory system and thermoregulation processes during and after efforts, undertaken in conditions of heat^2,3^ and cold^4^. It has also been researched how the type of exercise, its duration and intensity affect the process of the thermoregulation assessed on the basis of thermal radiation from the skin. In order to assess the efficiency of the circulatory system, measurements such as maximum/peak oxygen uptake (VO_2_max)^5^, heart rate recovery (HRR)^6^, exercise stroke volume (SV)^7^, sinus heart rate variability (HRV)^8^ are used. Previous articles have described the correlation between VO_2_max and changes in body surface temperature recorded during high-intensity exercise and those recorded during the recovery after high-intensity exercise^9,10^. The correlation of thermal parameters with heart rate recovery (HRR) and heart rate variability (HRV) recovery was also described^10^. Available reasearch^9,10^ indicates that body surface temperature measurement during exercise and recovery can be used to assess the efficiency of the circulatory system. There are two possible explanations for the above-described relationship between selected measures of cardiovascular fitness and the size of the thermal response to exercise. Firstly, an increase in the intensity of energy metabolism leads to increased production of thermal energy^11^. Energy metabolism in the efforts applied in the previous studies^9,10^ was largely dependent on the VO_2_max value, as the oxygen uptake in such efforts was recorded at a level close to VO_2_max^12,13^. Secondly, muscle and skin blood flow during exercise determines thermal energy transport to the body surface^14^, affecting the body surface temperature^12^. At the same time, muscle^15^ and skin^14^ blood flow during exercise are conditioned by the stroke volume, which is one of the determinants of VO_2_max^15^. Moreover, efficiently removing thermal energy may alleviate physiological stress and thus affect HRR and HRV recovery^10^.

So far^9^, thermal parameters measured at body surface were assessed in a test similar to sprint interval training, consisting of 4 efforts of 30 s each, performed at maximum intensity. Such a test leads to a significant disturbance of the acid–base balance^16^, and high production of phosphates and ammonia, which contribute to the rapid development of fatigue^17,18^. Therefore, this form of testing is very burdensome to the subjects. In addition, sprinting efforts such as the Wingate test may sometimes lead to syncope ^19^. For the reasons above, it was reasonable to look for a different research protocol to assess changes in body temperature induced by exercise. In subsequent studies, it was proposed to perform a more extended effort, lasting 3 min, with a power of 110% of the maximum power of the progressive test (110% Pmax)^10^. Such efforts lead to lower blood lactate levels^20^ compared to repeated sprints lasting 30 s each^14^. In addition, efforts performed with a power of 110% Pmax are used in performance diagnostics to verify peak oxygen uptake obtained in progressive tests^13,21^. However, the procedure described by Jastrzębska et al.^10^ has some shortcomings. First of all, it requires the subjects to make two visits 24 h apart in the laboratory, forcing them to submit to the guidelines during the entire research process (no stimulants, sleep time, etc.). Secondly, in this procedure, circulatory fitness is already assessed in the progressive test (GXT) on the basis of peak oxygen uptake, so performing an indirect assessment of circulatory fitness on the basis of thermal parameters in a test performed the next day seems to have little merit. In view of the above, it seems reasonable to seek an alternative procedure. Such a procedure should not require the prior use of a progressive test as a test to determine the intensity of effort for thermal performance.

Previous studies conducted by the team used high- and very high-intensity efforts. A sprint interval training test (4 × 30 s with maximum power)^9^ or a 3-min maximum effort with a power of 110% of Pmax determined in the progressive test are efforts intended for athletes. People who do not train, with low physical activity will not be able to properly perform the above-mentioned efforts due to too low muscle strength of the lower limbs and too rapid increase of fatigue^22^. Taking into account the fact that circulatory failure often affects people with lower physical activity and thus a lower level of physical efficiency, it seems right to look for a relationship between non-invasively measured thermal parameters from the body surface in response to efforts with submaximal intensities and parameters already used in the assessment of cardiovascular fitness.

In the search for the right test procedure, selecting the intensity of the effort is the key. It has been shown that maximum power in progressive exercise tests^23^, as well as oxygen uptake, and the intensity of energy metabolism during rest and submaximal work correlate with lean body mass^24^. Therefore, we decided that power in submaximal efforts shall be normalized to lean body mass. The study aimed to determine the strength of the relationship between selected measures of cardiovascular fitness and changes in body surface temperature recorded during light and heavy exercise of constant intensity normalized to lean body mass in young people. We assumed that exercise and recovery changes in body surface temperature would correlate with VO_2_max, Pmax and HRR.

## Material and methods

### Participants

25 non-smoking men aged 19–25 were recruited to participate in the study. Participants were enrolled into the study on the condition of participation in intensity spinning training. It was assumed that the participants would be excluded upon any threat to their health and wellbeing. In order to assess those:

- Medical history of each participant was gathered, with special emphasis of cardiovascular and respiratory system
- Resting blood pressure was measured before each effort test. It was assumed that individuals with diagnosed hypertension will be excluded from the effort tests. Hypertension was defined as the value of diastolic pressure over 90 mmHg and systolic pressure exceeding 140 mmHg, as reported by Schweiger et al.^25^. We accepted a margin of few mmHg because of the influence of stress on blood pressure^26^. Resting pulse rate was measured for each participant before taking the effort tests to exclude any individuals with tachycardia.

Additionally, each participant was required to take antigen test against COVID19 IgM. Any participant with positive results of such test were excluded from the study. The characteristics of the participants are presented in Table 1. All the subjects had participated in training on spinning bikes twice a week, for 60 min, for at least 6 months preceding the study. During that time, two types of trainings were undertaken. The first type consisted of HIIT trainings incorporating several maximum-effort trainings lasting 3–4 min, intermitted with active resting. The other type was threshold trainings consisted of high-intensity efforts close to functional threshold power (FTP), intermitted with active resting. Preliminary analysis of physical efficiency of the participants consisted of reviewing their FTP data, as defined in the procedure reported by Klitzke Borszcz et al.^27^ in 20-min maximal effort. The average FTP was 3.07 ± 0.59 W∙kg^−1^.

**Table 1:** Anthropometric characteristic of the group.

	Age [y]	Fat percentage [%]	Body high [m]	Body weight [kg]
Mean	21.5	14.9	1.80	77.6
Standard deviation	1.83	3.7	0.07	9.1
Lower 95% CI	20.7	13.4	1.77	73.9
Upper 95% CI	22.2	16.5	1.83	81.4

*CI* confidence interval.

### Test procedure

Before the study, the consent of the University Ethics Committee was obtained for implementing the project described in this work (Consent number: 39/2019). After the study procedures were explained, written informed consent has been obtained from all participants. The study consisted of three exercise tests. On the first day, a progressive test was performed, and then, after a 24-h break, a test verifying the VO_2_max value was performed, according to the previously described procedure^13^. After another 72 h, a test consisting of two submaximal efforts was performed. Study participants were instructed to have at least 9-h long night rest, abstain from alcohol, caffeine and intense physical effort for 48 h prior to the study and between each laboratory test. Participants were advised to intake at least 100 g carbohydrates in 3 h prior to the effort tests. In the course of effort tests and 48 h prior to the fist test, paricipants were asked to ingest 1.2–1.4g/kg of protein^28^, 3000 kcal^29^, and drink at least 2 l of water.

### Graded exercise test (GXT)

Prior to the graded exercise test, body composition was measured using near-infrared Futrex 6100/XL analyzer (Futrex, Hagerstown, USA). The analyzer head was applied to the anterior midline and in the midway of the *biceps brachii* of the dominant hand using the measure provided by the manufacturer. Then the measurement of the body fat content expressed in kilograms, the percentage of body weight, and lean body mass (LBM) was performed. The analyzer was calibrated prior to each measurement with the manufacturer-supplied optical standard. Before GXT and directly after the test, blood pressure was measured with aneroid sphygmomanometer (Riester, Germany). All measurements were taken by the same person to avoid any bias. Subsequently, initial and post-exercise stroke volume (SV) was measured with the following formula^30^:

$$SV=90.97+0.54\times PP-0.57\times DBP-0.61\times age$$

where SV—stroke volume (ml), PP—the difference between post-exercise systolic and diastolic blood pressure (mm Hg), DBP–post-exercise diastolic pressure (mm Hg).

The GXT was conducted on a Lode Excalibur Sport ergometer (Lode BV Groningen, Netherlands), calibrated before the study started. The effort began with a load of 50 W. Every 3 min, the load was increased by 50 W until the subject refused to continue the effort or until the pedaling rate fell below 60RPM. If a participant was unable to complete an entire 3 min stage, 0.28 W per second missed was subtracted from the work rate at that stage. The highest power output determined in the GXT was taken to be the measure of maximal aerobic power (Pmax). During the test, respiratory parameters were measured using a Quark CPET ergo spirometer (Cosmed, Milan, Italy). The ergo spirometer was calibrated before testing with a standard gas mixture of 5% carbon dioxide, 16% oxygen, and 79% nitrogen. Breathing air was analyzed breath-by-breath to measure oxygen uptake (VO_2_) and carbon dioxide excretion (VCO_2_). The measurements were then averaged over 30-s intervals. The highest oxygen uptake value obtained was considered the peak oxygen uptake in the GXT (VO_2_peak1).

VO_2_max value was verified with a test with a power of 110% Pmax following the previously described procedure^13^ on the day following the GXT. As in the graded exercise test, respiratory parameters were measured during the verification test. The parameters were then averaged in 30-s intervals. The highest oxygen uptake value obtained in the verification test was considered the peak oxygen uptake of this test (VO_2_peak2). Subsequently, the higher value of VO_2_peak1 and VO_2_peak2 was considered the maximum oxygen uptake (VO_2_max).

**Figure 1:**
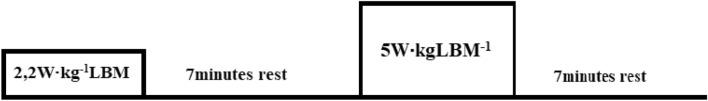
Diagram showing the course of a constant-intensity exercise test.

The constant-intensity exercise test consisted of two exercises lasting 3 min each and was performed with an intensity of 2.2 W∙kg^−1^ of lean body mass (2.2 W∙kgLBM^−1^) and 5 W∙kgLBM^−1^, respectively. The intensity of the efforts was selected in W∙kgLBM^−1^ because muscle power depends on lean body mass^31^. By normalizing the load considering LBM, we intended to obtain similar work intensity for each subject. The load sizes were selected based on the data from the GXT. In the GXT we did not observe an increase in lactate concentration above 2 mmol l^−1^, nor an increase in VE∙VO_2_^–1^ equivalent in any of the subjects up to an intensity of ≈ 2.2 W∙kgLBM^−1^. Therefore, based on the criteria described by MacIntosh et al.^32^, we considered an effort of 2.2 W∙kgLBM^−1^ to be a low-intensity effort. We then determined that at an intensity of ≈ 5 W∙kgLBM^−1^, none of the subjects was able to continue the GXT. Therefore, we considered the intensity of 5 W∙kgLBM^−1^ as a heavy effort. Each of the efforts mentioned above was followed by a passive break lasting 7 min. Figure 1 shows the scheme of the submaximal efforts test.

During the constant-intensity exercise test, the density of thermal radiation from the body surface was measured using a Sonel KT384 thermal imaging camera (Sonel SA, Świdnica, Poland). The camera has a resolution of IR 384 × 288, a spectral range of 8^−1^4 μm, thermal sensitivity of 0.08 ℃. The software provided by the manufacturer (Sonel ThermoAnalyze) converted the radiation density into body surface temperature expressed in ℃. During the playback of the recorded video, individual frames were analyzed. The average temperature was recorded within a square field (10 pixels on a side), marked individually on the temple, as in previous publications^9,10^. The center of the thermal analysis field was located halfway between the hairline and the end of the eyebrow arch. The lower edge of the analysis field was located at the height of the lateral end of the brow arch. Immediately before the start of each test effort, the initial temperature of the temple surface was indicated (T-2.2_baseline_, T-5_baseline_). Subsequent measurements of the temple surface temperature were made immediately after each test effort (T-2.2_end_, T-5_end_) and after 2 min of recovery after each test effort (T-2.2_rest,_ T-5_rest_). Then, the differences between T-…_end_ and T-…_baseline_ were calculated for each test effort (ΔT1-2.2; ΔT1-5, respectively) and the differences between T-…_rest_ and T-…_end_ (ΔT2-2.2; ΔT2-5, respectively), similar to the calculations made by Jastrzębska et al.^10^.

The heart rate was recorded using a V800 cardiofrequency meter (Polar, Oy, Finland). Based on the heart rate recording, the contraction rates were indicated for 1′ (HRR1′), 2′ (HRR2′), 3′ (HRR3′), 4′ (HRR4′), 5′ (HRR5′) and 6′ (HRR6′) minutes of restitution after each test effort. Then, changes in heart rate during recovery were calculated as the difference between the heart rate measured in the final phase of each test effort and the corresponding recovery value: HRR1′ (ΔHRR1′), HHR2′ (ΔHRR2′), HRR3′ (ΔHRR3′), HRR4 (ΔHRR4′), HRR5′ (ΔHRR5′), HRR6′ (ΔHRR6′), similar to Suzic Lazic et al.^33^.

### Statistical analysis

Statistical analysis and visualisations of the data were prepared using Statistica 13 software (Statsoft Polska, Cracow, Poland). The Shapiro–Wilk W test was used to evaluate the measured data distribution. Only post-exercise SV normalized to body weight and LBM differed significantly from the normal distribution. Repeated measures analysis of variance was used to assess the differences between T-…_baseline,_ T-…_end,_ T-…_rest_ at each intensity of the submaximal exercise test.

Strength of the relationship between changes in the temple surface temperature and measures of cardiovascular fitness (and physical efficiency) was determined using the simple Pearson correlation. For the data statistically different from normal distribution Spearman rank test was used.

Using the formula for the critical value of the correlation coefficient, the minimum group size was calculated, assuming that the acceptable level of statistical significance (α) is 0.05 and the correlation strength should be very high (r ≥ 0.7). The following formula was used:

$$r=\sqrt{\frac{{t}_{\alpha }^{2}}{n-2+{t}_{\alpha }^{2}}}$$

Based on this, we determined that the minimum number of participants is 8 at t ≈ 2.44.

### Institutional review board statement

The study was conducted according to the guidelines of the Declaration of Helsinki, and approved by Ethics Committee of the University School of Physical Education in Wroclaw (protocol code: 39/2019; date of approval: 26 November 2019).

## Results

Table 2 presents the basic statistics of the analyzed variables. The effect of repeated measurements on the temple surface temperature during exercise with a power of 2.2 W∙kgLBM^−1^ was not detected. The effect of repeated measurements was demonstrated for the temperature of the temple surface during exercise with a power of 5 W∙kgLBM^−1^ (F = 12.70; η^2^ = 0.35; *p* = 0.00). A post hoc test revealed a statistically significant difference between T-5_end_ and either T-5_baseline_ or T-5_rest_.

**Table 2:** Basic statistics of the variables included in the data analysis.

Parameters	Mean	Standard deviation	Lower 95%CI	Upper 95%CI
VO_2_max [l∙min^−1^]	3.87	0.38	3.71	4.03
VO_2_max [ml∙kg^−1^∙min^−1^]	50.37	6.88	47.54	53.21
VO_2_max∙LBM^−1^[ml∙kg^−1^∙min^−1^]	59.11	6.55	56.41	61.82
Pmax [W]	297.72	40.74	280.90	314.54
Pmax [W∙kg^−1^]	3.87	0.66	3.60	4.15
Pmax∙LBM^−1^ [W∙kg^−1^]	4.54	0.63	4.28	4.80
SV_baseline_ [ml]	62.48	8.69	58.89	66.06
SV_exercise_ [ml]	118.56	21.70	109.60	127.51
SV_baseline_ [ml∙kg^−1^]	0.82	0.16	0.75	0.88
SV_exercise_ [ml∙kg^−1^]	1.54	0.31	1.41	1.67
SV∙LBM_baseline_ [ml∙kg^−1^]	0.98	0.16	0.89	1.02
SV∙LBM_exercise_ [ml∙kg^−1^]	1.81	0.34	1.67	1.95
T-2.2_baseline_ [°C]	32.78	2.31	31.83	33.74
T-2.2_end_ [°C]	32.54	2.62	31.45	33.62
T-2.2_rest_ [°C]	33.20	3.06	31.93	34.46
T-5_baseline_ [°C]	30.09	1.59	29.43	30.74
T-5_end_ [°C]	29.36*	1.92	28.56	30.15
T-5_rest_ [°C]	30.51^	1.57	29.86	31.16

*VO*_*2*_*max* maximum oxygen uptake; *LBM* lean body mass; *Pmax* maximum aerobic power in GXT; *SV* stroke volume measured before GXT (baseline) and immediately after GXT (exercise); *T* temporal temperature measured immediately before the effort test (_baseline_), immediately after the effort test (_end_) and during the 2 min of recovery (_recovery_); *p < 0.05 versus T…_baseline_; _._ ^*p* < 0.05 versus T…_end_.

**Figure 2:**
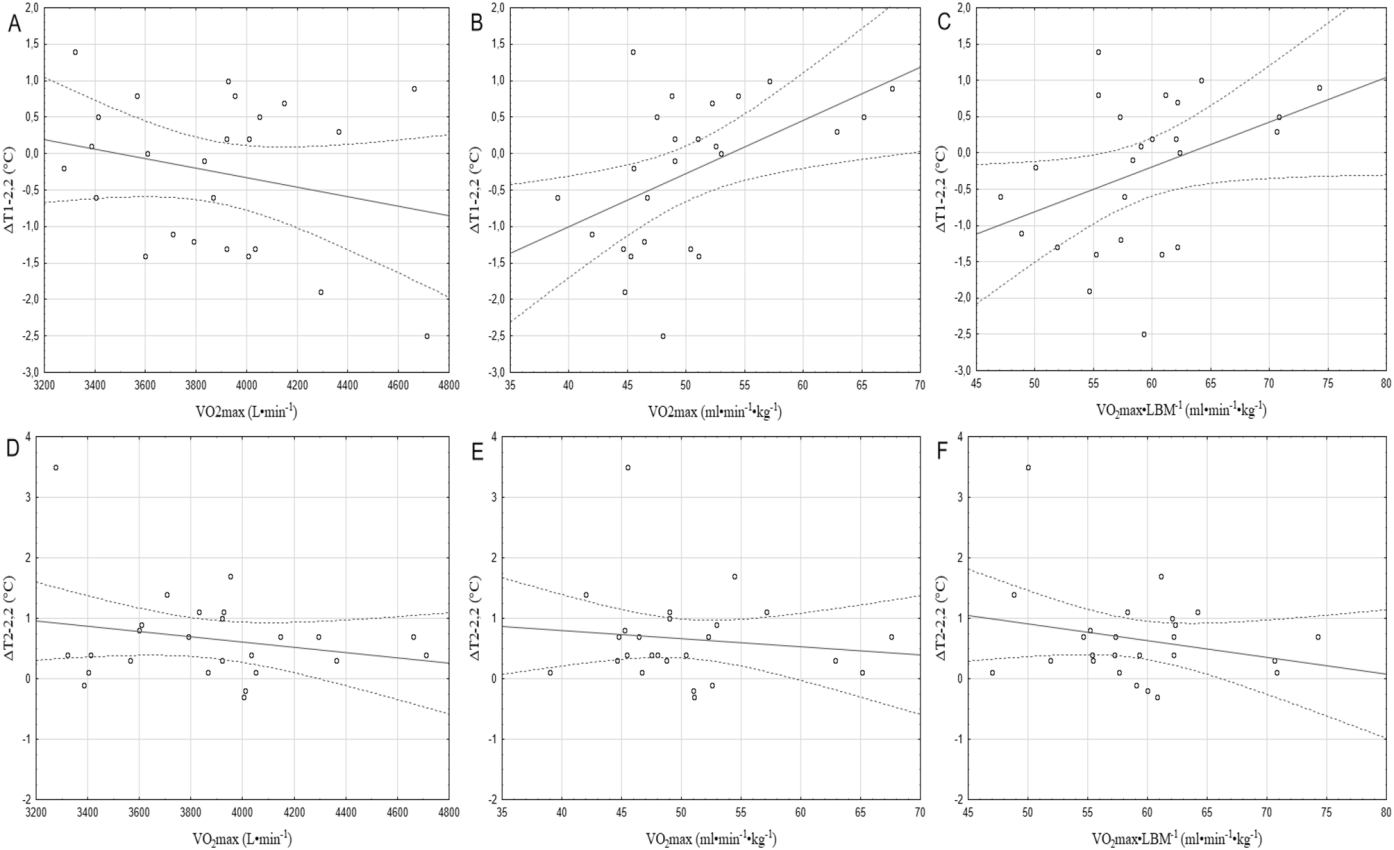
Pearson’s correlation plots between maximal oxygen uptake and changes in body surface temperature during exercise with an intensity of 2.2 W·kgLBM^−1^.

**Figure 3:**
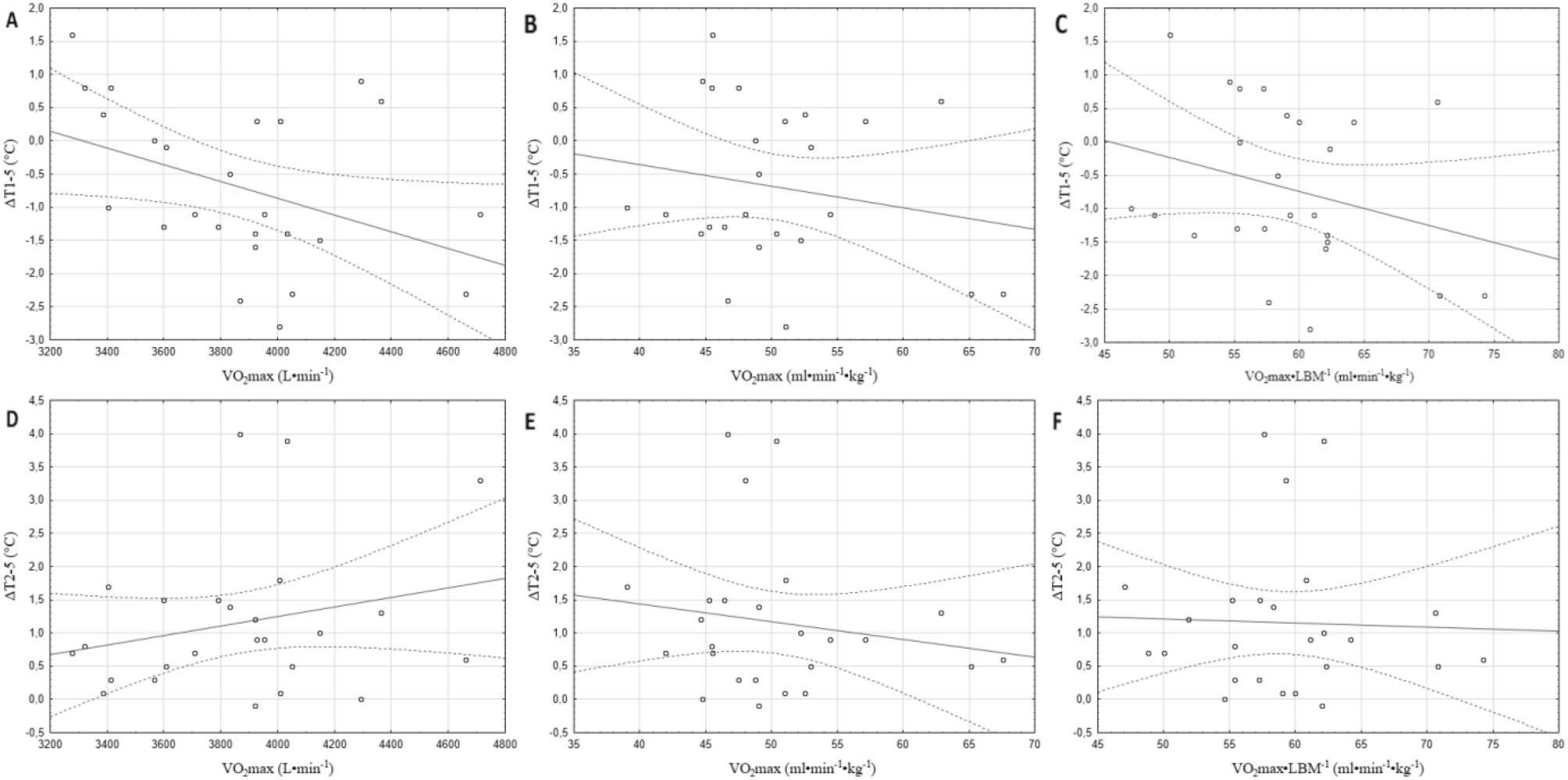
Pearson’s correlation plots between maximal oxygen uptake and body surface temperature changes during 5 W·kgLBM^−1^ intensity exercise.

A statistically significant correlation (r = 0.49; *p* < 0.05) was found between ΔT1-2.2 and VO_2_max expressed in ml min^−1^ kg^−1^ (Fig. 2B). The correlations between ΔT1-2.2 and the other parameters (Fig. 2A,D,E,F) were trivial (r = 0.1–0.3) or moderate (Fig. 2C) (r > 0.03) and statistically insignificant. In the analysis of the effort with an intensity of 5 W∙kgLBM^−1^, a statistically significant correlation (r = − 0.41; *p* < 0.05) was found only between ΔT1-5 and VO_2_max expressed in min^−1^ (Fig. 3A). The correlations with other parameters were insignificant with level of strength weak (r < 0.1; Fig. 3F), trivial (r = 0.1–0.3; Fig. 3B–E).

Baseline SV normalized to body weight and LBM correlated with ΔT1-2.2 (Table 3). In the analyses concerning the GXT maximum power, a statistically significant correlation was found only between ΔT1-2.2 and Pmax expressed in W kg^−1^. In addition, a correlation with the required threshold of statistical significance was found between HRR1’ and ΔT2-2.2 and between HRR3’ and ΔT1-5 (Table 3).

**Table 3:** Pearson correlation values between thermal parameters and the maximum power of the GXT, and between thermal parameters and heart rate recovery.

	ΔT1-2.2	ΔT1-5	ΔT2-2.2	ΔT2-5
SV_baseline_ [ml]	0.05	0.23	− 0.06	− 0.01
SV_exercise_ [ml]	− 0.23	− 0.13	− 0.15	− 0.01
SV_baseline_ [ml kg^−1^]	0.51*	0.25	− 0.03	− 0.25
SV_exercise_ [ml kg^−1^]	0.11	− 0.05	0.12	− 0.26
SV∙LBM_baseline_ [ml∙kg^−1^]	0.45*	0.26	− 0.10	− 0.17
SV∙LBM_exercise_ [ml kg^−1^]	− 0.01	− 0.09	0.07	− 0.18
Pmax [W]	− 0.18	− 0.31	− 0.13	0.07
Pmax [W∙kg^−1^]	0.41*	− 0.18	− 0.08	− 0.22
Pmax∙LBM^−1^ [W∙kg^−1^]	0.33	− 0.26	− 0.16	− 0.14
HRR1′ [bpm]	− 0.28	− 0.10	− 0.43*	0.10
ΔHRR1′ [bpm]	− 0.12	− 0.01	0.28	− 0.09
HRR2′ [bpm]	− 0.21	− 0.28	− 0.25	0.06
ΔHRR2′ [bpm]	− 0.16	0.24	0.11	− 0.01
HRR3′ [bpm]	− 0.30	− 0.40*	− 0.28	0.03
ΔHRR3′ [bpm]	0.00	0.36	0.13	0.04
HRR4′ [bpm]	− 0.25	− 0.30	− 0.30	− 0.01
ΔHRR4′ [bpm]	− 0.11	0.24	0.08	0.09
HRR5′ [bpm]	− 0.23	− 0.29	− 0.30	0.01
ΔHRR5′ [bpm]	− 0.09	0.22	0.13	0.08
HRR6′ [bpm]	− 0.17	− 0.34	− 0.24	0.03
ΔHRR6′ [bpm]	− 0.19	0.27	0.05	0.04

*ΔT1* difference between temporal temperature measured immediately after the constant-intensity effort and baseline temporal temperature measured immediately before the start of constant-intensity effort; *ΔT2* difference between temporal temperature measured after 2 min of recovery and immediately after the constant intensity effort; *SV* stroke volume measured before GXT (baseline) and immediately after GXT (exercise); *Pmax* maximal power measured in GXT; *LBM* lean body mass; *HRR* heart rate recovery measured in 1, 2, 3, 4, 5, 6 min of recovery after a constant-intensity effort; *ΔHRR* difference between heart rate measured at the end of the effort and the heart rate measured at the end of 1, 2, 3, 4, 5, 6 min of recovery; 1′, 2′ 3′, 4′, 5′, 6′—measurements taken in the next minutes of recovery after the efforts; *p < 0.05.

## Discussion

It is assumed that during short-term and intense exercise, skin blood flow decreases due to vasoconstriction, which has been described by several authors^34–36^. The decreasing volume of skin blood flow causes the temperature of the body surface to decrease during short-term and intense exercise performed at the ambient temperature of approx. 20 °C^2^. Also, the studies described in this paper showed that the body surface temperature decreased immediately after the exercise with the power of 5 W∙kgLBM^−1^. Comparative studies revealed differences between athletes and untrained people in the dynamics of exercise changes in body surface temperature^37,38^. Moreover, negative correlations were found between selected parameters of cardiovascular fitness (and physical capacity) and thermal responses to exercise^9,10^. The results of these correlations indicated that high efficiency of the circulatory system is associated with a relatively significant decrease in temple temperature during exercise. The data presented herein confirm the previous findings, as we obtained a statistically significant negative correlation between ΔT1-5 and VO_2_max expressed in absolute values.

The statistical analysis results for the data recorded during exercise with a power of 2.2 W∙kgLBM^−1^ indicate a positive correlation between ΔT1-2.2 and Pmax normalized to body weight and a positive correlation between ΔT1-2.2 and VO_2_max normalized to body weight. In addition, a positive correlation between ΔT1-2.2 and SV_baseline_ normalized to body weight and normalized to LBM was demonstrated. The direction of this correlation is opposite to the compounds described by Hebisz et al.^9^, Jastrzębska et al.^10^ and the relationships described above regarding the effort with the power of 5 W∙kgLBM^−1^. We believe that the reason for obtaining the opposite direction of correlation for the data collected during exercise with the power of 2.2 W∙kgLBM^−1^ is the use of lower intensity of the test effort compared to Hebisz et al.^9^ and Jastrzębska et al.^10^. As mentioned above, during high-intensity exercise, in its initial phase, body surface temperature decreases^2^. However, if the exercise is of low intensity, in its initial phase, the decrease in body surface temperature is smaller than during intense exercise^2^. These differences may be due to different blood flow patterns depending on the intensity of exercise performed^12,36^. In addition, during light exercise, after a temporary decrease, blood flow begins to increase^36^, which can increase body surface temperature. The correlation results presented in this paper indicate that the magnitude of the increase in body surface temperature during low-intensity exercise is related to the level of aerobic capacity.

When exercise is short-term and intense, the body surface temperature measured immediately after exercise is lower than that measured before exercise^10^. Then, a significant increase in body surface temperature is observed during the recovery after exercise^9,10^. It was also shown that recovery changes in temple temperature after intense exercise strongly and positively correlated with measures of cardiovascular fitness and aerobic physical efficiency^9,10^. This study showed that the change in body surface temperature was statistically significantly correlated only with HRR1 after exercise with an intensity of 2.2 W kgLBM^−1^. Therefore, the data presented in this paper indicate that the relationship between ΔT2 and the applied measures of physical capacity is insignificant. The only correlation that reached the required level of statistical probability concerns the value of the heart rate measured at restitution. This is a negligible effect because it was assumed that heart rate recovery is strongly related to cardiovascular fitness when analyzed as ΔHRR^39,40^. Therefore, the analysis of ΔT2 after exercise with a power less than or equal to 5 W∙kgLBM^−1^ is not an efficient way to assess the efficiency of the cardiovascular system.

The correlations presented in this work indicate that approx. 17–24% of exercise capacity (VO_2_max or Pmax) can be explained by exercise thermal responses to efforts less than or equal to 5 W∙kgLBM^−1^. Earlier studies, which used exercise tests of greater intensity, exceeding the power of the progressive test, achieved greater correlation strength^9,10^ however they were performed by cyclists, characterized by higher physical capacity. Therefore, we believe that the test procedure used in this study does not allow for accurate diagnosis of cardiovascular fitness or aerobic physical capacity using ΔT1 or ΔT2 in efforts performed with an intensity of up to 5 W∙kgLBM^−1^. For diagnostic purposes, it may be better to measure changes in body surface temperature during efforts of greater intensity than 5 W∙kgLBM^−1^, as was done in the study by Hebisz et al.^9^ and Jastrzębska et al.^10^.

Limitations. The main reasoning behind this study is demonstrating the relationship between post-exercise changes in body surface temperature and measures of cardiovascular fitness. Due to the low power of the relationships described in this study is moderate, further research may be warranted to eliminate the methodological limitations of our research. The study sample of 25 men is relatively small, which makes it difficult to generalize the conclusions resulting from the research performed. Therefore, research planned in the future should include the recruitment of a larger research group. Moreover, we couldn’t control if participants implemented our recommendations on the amount of sleep and the principles of nutrition during the study period. This is a significant limitation of our research, as sleep has a considerable impact on restitution processes such as muscle regeneration, nervous system and immune system^41^, and sleep deprivation impairs athletes’ performance^42^. Therefore, future studies with short time between exercise tests will have to implement monitoring of the quality of sleep.

## Conclusions.

Changes in body surface temperature in response to exercise positively correlate with measures of aerobic exercise capacity, such as VO_2_max and Pmax, when exercise is of moderate intensity. When the exercise intensity is high (5 W kgLBM^−1^), the correlation between exercise body temperature change and VO_2_max becomes negative. These results indicate that the relationship between body surface temperature and aerobic physical performance depends on the intensity of the test effort. The strength of these correlations is too low to be used to determine aerobic exercise capacity based on temporal thermal radiation. Other papers have found correlations at the level of ≈ 0.8–0.9 between VO_2_max and submaximal VO_2_ measured at the anaerobic threshold or at the respiratory compensation point^43^ or correlations at the level of ≈ 0.9 between VO_2_max and HRmax∙HRrest^−1^^44^ among people with a similar level of performance to our research group. Finally, futer studies shoul investigate the potential role of thermoregulatory efficiency in the relationshoi between body surface temperature and aerobic exercise capacity.

## Data availability

The data presented in this study are available on request from the corresponding author.

## References

[1] Raghuveer G (2020). American Heart Association young hearts athero, hypertension and obesity in the young committee of the council on lifelong congenital heart disease and heart health in the young. Cardiorespiratory fitness in youth: An important marker of health: A scientific statement from the American Heart Association. Circulation.

[2] Périard JD, Eijsvogels TMH, Daanen HAM (2021). Exercise under heat stress: thermoregulation, hydration, performance implications, and mitigation strategies. Physiol. Rev..

[3] Kenney WL, Wolf ST, Dillon GA, Berry CW, Alexander LM (2021). Temperature regulation during exercise in the heat: Insights for the aging athlete. J. Sci. Med. Sport.

[4] Chen F, Fu M, Li Y, Shen S, Guo X (2022). Modelling and experimental study of thermo-physiological responses of human exercising in cold environments. J. Thermal Biol..

[5] Hansen MT (2023). Accuracy of a clinical applicable method for prediction of VO_2_max using seismocardiography. Int. J. Sports Med..

[6] Fan LM, Collins A, Geng L, Li JM (2020). Impact of unhealthy lifestyle on cardiorespiratory fitness and heart rate recovery of medical science students. BMC Public Health.

[7] Nystoriak MA, Bhatnagar A (2018). Cardiovascular effects and benefits of exercise. Front. Cardiovasc. Med..

[8] Souza HCD, Philbois SV, Veiga AC, Aguilar BA (2021). Heart rate variability and cardiovascular fitness: What we know so far. Vasc. Health. Risk. Manag..

[9] Hebisz R, Hebisz P, Borkowski J, Wierzbicka-Damska I, Zatoń M (2019). Relationship between the skin surface temperature changes during sprint interval testing protocol and the aerobic capacity in well-trained cyclists. Physiol. Res..

[10] Jastrzębska AD, Hebisz R, Hebisz P (2022). Temporal skin temperature as an indicator of cardiorespiratory fitness assessed with selected methods. Biology (Basel)..

[11] Kenny GP, McGinn R (2017). Restoration of thermoregulation after exercise. J. Appl. Physiol..

[12] Hebisz R, Hebisz P, Zatoń M, Michalik K (2017). Peak oxygen uptake in a sprint interval testing protocol vs. maximal oxygen uptake in an incremental testing protocol and their relationship with cross-country mountain biking performance. Appl. Physiol. Nutr. Metab..

[13] Hebisz P, Jastrzębska AD, Hebisz R (2021). Real assessment of maximum oxygen uptake as a verification after an incremental test versus without a test. Front. Physiol..

[14] Low DA, Jones H, Cable NT, Alexander LM, Kenney WL (2020). Historical reviews of the assessment of human cardiovascular function: interrogation and understanding of the control of skin blood flow. Eur. J. Appl. Physiol..

[15] Joyner MJ, Casey DP (2015). Regulation of increased blood flow (hyperemia) to muscles during exercise: A hierarchy of competing physiological needs. Physiol. Rev..

[16] Hebisz R, Hebisz P, Borkowski J, Zatoń M (2016). Differences in physiological responses to interval training in cyclists with and without interval training experience. J. Hum. Kinet..

[17] Ulupınar S (2021). Effects of sprint distance and repetition number on energy system contributions in soccer players. J. Exerc. Sci. Fit..

[18] Morcillo JA (2015). Relationships between repeated sprint ability, mechanical parameters, and blood metabolites in professional soccer players. J. Strength Cond. Res..

[19] Horiuchi M, Nishida A, Dobashi S, Koyama K (2022). Comparisons between normobaric normoxic and hypoxic recovery on post-exercise hemodynamics after sprint interval cycling in hypoxia. Front. Physiol..

[20] Messonnier LA, Chatel B, Emhoff CW, Blervaque L, Oyono-Enguéllé S (2021). Delayed rebound of glycemia during recovery following short-duration high-intensity exercise: Are there lactate and glucose metabolism interactions?. Front. Nutr..

[21] Follador L (2018). Physiological, perceptual, and affective responses to six high-intensity interval training protocols. Percept. Mot. Skills..

[22] Villanueva IR (2021). Comparison of constant load exercise intensity for verification of maximal oxygen uptake following a graded exercise test in older adults. Physiol. Rep..

[23] Maciejczyk M, Wiecek M, Szymura J, Szygula Z, Brown LE (2015). Influence of increased body mass and body composition on cycling anaerobic power. J. Strength Cond. Res..

[24] Pourhassan M (2015). Relationship between submaximal oxygen uptake, detailed body composition, and resting energy expenditure in overweight subjects. Am. J. Hum. Biol..

[25] Schweiger V, Niederseer D, Schmied C, Attenhofer-Jost C, Caselli S (2021). Athletes and hypertension. Curr. Cardiol. Rep..

[26] Wright BJ, O'Brien S, Hazi A, Kent S (2014). Increased systolic blood pressure reactivity to acute stress is related with better self-reported health. Sci. Rep..

[27] Klitzke Borszcz F, Tramontin AF, Costa VP (2020). Reliability of the functional threshold power in competitive cyclists. Int. J. Sports Med..

[28] Kato H, Suzuki K, Bannai M, Moore DR (2016). Protein requirements are elevated in endurance athletes after exercise as determined by the indicator amino acid oxidation method. PLoS ONE.

[29] Burke LM, Hawley JA, Wong SH, Jeukendrup AE (2011). Carbohydrates for training and competition. J. Sports Sci..

[30] Mokasheva EN (2023). Rapid assessment of cardiovascular system parameters using cardiorespiratory indices. Biol. Bull. Rev..

[31] Coelho-E-Silva MJ (2020). Allometric modeling of Wingate test among adult male athletes from combat sports. Medicina (Kaunas).

[32] MacIntosh BR, Murias JM, Keir DA, Weir JM (2021). What is moderate to vigorous exercise intensity?. Front. Physiol..

[33] Suzic Lazic J (2017). Heart rate recovery in elite athletes: The impact of age and exercise capacity. Clin. Physiol. Funct. Imaging.

[34] Johnson JM (1992). Exercise and the cutaneous circulation. Exerc. Sport Sci. Rev..

[35] Kamon E, Belding HS (1969). Dermal blood flow in the resting arm during prolonged leg exercise. J. Appl. Physiol..

[36] Simmons GH (2011). Increased brachial artery retrograde shear rate at exercise onset is abolished during prolonged cycling: Role of thermoregulatory vasodilation. J. Appl. Physiol. (1985).

[37] Formenti D (2013). Thermal imaging of exercise-associated skin temperature changes in trained and untrained female subjects. Ann. Biomed. Eng..

[38] Galan-Carracedo J, Suarez-Segade A, Guerra-Balic M, Oviedo GR (2019). The dynamic and correlation of skin temperature and cardiorespiratory fitness in male endurance runners. Int. J. Environ. Res. Public. Health..

[39] Hirsh DS (2006). Association of heart rate recovery and maximum oxygen consumption in patients with chronic congestive heart failure. J. Heart Lung Transplant..

[40] Pakkala A, Veeranna N, Kulkarni SB (2005). A comparative study of cardiopulmonary efficiency in athletes and non-athletes. J. Indian Med. Assoc..

[41] Taheri M, Valayi F (2019). Aerobic exercise improves attention and quality of sleep among professional volleyball players. Sleep Hypn..

[42] Paryab N, Taheri M, Irandoust K, Mirmoezzi M (2020). Effects of melatonin on neurological function and maintenance of physical and motor fitness in collegiate student-athletes following sleep deprivation. Int. J. Sport Stud. Health..

[43] Wiecha S (2023). VO_2_max prediction based on submaximal cardiorespiratory relationships and body composition in male runners and cyclists: A population study. Elife.

[44] Uth N, Sørensen H, Overgaard K, Pedersen PK (2004). Estimation of VO_2_max from the ratio between HRmax and HRrest–the Heart Rate Ratio Method. Eur. J. Appl. Physiol..

